# Genetic Improvement of *Torulaspora delbrueckii* for Wine Fermentation: Eliminating Recessive Growth-Retarding Alleles and Obtaining New Mutants Resistant to SO_2_, Ethanol, and High CO_2_ Pressure

**DOI:** 10.3390/microorganisms8091372

**Published:** 2020-09-07

**Authors:** Rocío Velázquez, Alberto Martínez, Emiliano Zamora, María L. Álvarez, Joaquín Bautista-Gallego, Luis M. Hernández, Manuel Ramírez

**Affiliations:** 1 Departamento de Ciencias Biomédicas (Área de Microbiología), Facultad de Ciencias, Universidad de Extremadura, 06006 Badajoz, Spain; rociovelazquez1981@gmail.com (R.V.); amartinetp@alumnos.unex.es (A.M.); joaquin.bautistagallego@gmail.com (J.B.-G.); lmhernan@unex.es (L.M.H.); 2 Estación Enológica, Junta de Extremadura, 06200 Almendralejo, Spain; emiliano.zamora@juntaex.es (E.Z.); luz.alvarez@juntaex.es (M.L.Á.)

**Keywords:** *Torulaspora delbrueckii*, wine fermentation, spore clone, sparkling wine, ethanol resistance, SO_2_ resistance, pressure resistance

## Abstract

The use of *Torulaspora delbrueckii* has been repeatedly proposed to improve a wine’s organoleptic quality. This yeast has lower efficiency in completing wine fermentation than *Saccharomyces cerevisiae* since it has less fermentation capability and greater sensitivity to SO_2_, ethanol, and CO_2_ pressure. Therefore, the completion of fermentation is not guaranteed when must or wine is single-inoculated with *T. delbrueckii*. To solve this problem, new strains of *T. delbrueckii* with enhanced resistance to winemaking conditions were obtained. A genetic study of four wine *T. delbrueckii* strains was carried out. Spore clones free of possible recessive growth-retarding alleles were obtained from these yeasts. These spore clones were used to successively isolate mutants resistant to SO_2_, then those resistant to ethanol, and finally those resistant to high CO_2_ pressure. Most of these mutants showed better capability for base wine fermentation than the parental strain, and some of them approached the fermentation capability of *S. cerevisiae*. The genetic stability of the new mutants was good enough to be used in industrial-level production in commercial wineries. Moreover, their ability to ferment sparkling wine could be further improved by the continuous addition of oxygen in the culture adaptation stage prior to base wine inoculation.

## 1. Introduction

Among non-*Saccharomyces* yeasts, *Torulaspora delbrueckii* is probably the one with a wine-fermentation performance closest to *Saccharomyces cerevisiae*, and therefore the most suitable for winemaking. This yeast has recently been recommended for must fermentation mainly because it improves some wine parameters, such as decreased acetic acid and ethanol production, increased amount of glycerol, increased mannoprotein and polysaccharide release, promotion of malolactic fermentation, increased amounts of wanted aromatic compounds (fruity esters, lactones, thiols, and terpenes), and decreased amounts of unwanted aromatic compounds (such as higher alcohols). The features of this yeast may improve wine quality or complexity (reviewed in [1,2]). In addition, it may stimulate malolactic fermentation [3]. However, *T. delbrueckii* has some serious drawbacks that may discourage its use in winemaking. It has higher rates of CO_2_ production and O_2_ consumption than *S. cerevisiae*, which result in low biomass yield from batch cultures and is a handicap for the commercial production of *T. delbrueckii* [4]. Although both yeasts have very similar patterns for sugar utilization under fully respiratory growth conditions, *T. delbrueckii* grows more slowly than *S. cerevisiae* under strict anaerobic conditions [5,6]. As a consequence, *T. delbrueckii* has less fermentation vigor than *S. cerevisiae* under usual wine fermentation conditions, and has serious difficulties in dominating wine fermentation even when initially inoculated at a high proportion (above 10^7^ CFU/mL) [2,4,7]. These drawbacks are especially serious when making white and sparkling wines, which is usually done under strict anaerobic conditions [2]. Besides this, *T. delbrueckii* is also less resistant to other stressing conditions closely related to winemaking than *S. cerevisiae*, such as the rapid increase of ethanol concentration, the presence of SO_2_, and high CO_2_ pressure. These circumstances negatively affect the fermentation efficiency of *T. delbrueckii* during still or sparkling wine making. As this yeast has poor resistance to the fast increase of ethanol concentration, its fermentation rate slows down and cell death increases once the tumultuous fermentation stage of sugar-rich substrates has been reached [8]. As a consequence, fermentation may slow to become sluggish or even stop, or it may continue because of the participation of contaminating wild *Saccharomyces* yeasts [7,9]. Such a sequence of events reduces the participation of *T. delbrueckii* during must fermentation, making the real influence of this yeast on wine composition and quality uncertain [1]. The difference in ethanol resistance between *T. delbrueckii* and *S. cerevisiae* is easily visible on Yeast extract-Peptone-Dextrose growth medium (YEPD) plates supplemented with different ethanol concentrations [2]. Nevertheless, some strains of *T. delbrueckii* can dominate and complete crushed grape fermentation under specific favorable conditions (low amounts of competitor *S. cerevisiae* yeasts, large inoculum of healthy *T. delbrueckii* cells, frequent shaking to provide extra oxygen, addition of extra amounts of nutrients, and low amount of SO_2_) to reach greater than 14% ethanol concentration [2,10]. Even under these favorable conditions, *T. delbrueckii* fermentation takes much longer to complete than *S. cerevisiae* fermentation. However, as the rate of rise of ethanol production is rather low, part of the *T. delbrueckii* population has a chance to progressively become adapted to the stressing conditions and be able to complete grape fermentation.

As for ethanol, SO_2_ is another important stress factor since this compound is used during winemaking as a microbial inhibitor and antioxidant. Therefore, SO_2_ resistance is a desired trait for wine yeast strains [11]. The lower SO_2_ resistance of *T. delbrueckii* with respect to *S. cerevisiae* is clearly visible on Synthetic Defined medium (SD) agar plates supplemented with different concentrations of this compound. Despite this, *T. delbrueckii* is able to complete fermentation in the presence of 50 mg/L SO_2_. However, a concentration of 125 mg/L SO_2_, as is frequently used in winemaking, is lethal for *T. delbrueckii* [2].

Recently, trials using *T. delbrueckii* for sparkling wine making have been carried out. Some strains of this yeast can be inoculated in the first-fermentation of grape must to obtain base wine with improved quality [7,12]. However, these strains were unable to complete the second-fermentation of the base wine because they cannot survive above 3.5 atm of CO_2_ pressure inside a glass bottle of sparkling wine [12]. As a consequence, base wine single-inoculated with *T. delbrueckii* only completed second-fermentation when there were contaminant *Saccharomyces* yeasts, which became dominant after CO_2_ pressure rose above 3 atm, while *T. delbrueckii* progressively became inviable [2]. Therefore, high CO_2_ pressure is another factor that decreases the competitiveness of *T. delbrueckii* compared to its potential competitor *S. cerevisiae*. This suggests that its resistance to high pressure should be improved if any dominance of *T. delbrueckii* during second fermentation of sparkling wine is intended.

Based on our experience, the isolation of genetically stable mutants resistant to high ethanol concentrations is not an easy task. It is possible to isolate *S. cerevisiae* clones that are resistant to as much as 19% ethanol. However, these clones seem to be yeasts that adapt slowly to growth in these stressing conditions, and do not maintain the ethanol resistance phenotype once they are newly grown in the absence of ethanol and put back into media with high ethanol concentrations. Although changes of expression in a single gene may generate the tolerant phenotype [13,14], this occurs with difficulty since the tolerance to stressors requires changes of expression for many genes [15]. Besides this, yeast tolerance to high ethanol concentrations is temperature dependent since both factors exert a synergistic negative effect on yeast growth and enzymes [16]. Additionally, other environmental factors such as the nutrients available, osmotic pressure, or the way the carbohydrate substrate is added (sequentially, or all at the time of yeast inoculation) may also dramatically influence yeast ethanol tolerance [17,18,19]. Increasing the SO_2_ tolerance in yeast may also interfere with basic cellular metabolism and involve an interplay of genetic changes. Although some yeasts, including *S. cerevisiae*, have developed physiological mechanisms for SO_2_ tolerance, some of these molecular mechanisms are also complex and have only recently been investigated. Among them are sulfite reduction, sulfite oxidation, acetaldehyde production, sulfite efflux, and behaving as viable but not culturable cells [20]. In addition, SO_2_ tolerance has been connected to a molecular mechanism which involves a higher transcription level of the *SSU1* gene [21,22,23]. The molecular mechanisms involved in CO_2_ pressure tolerance have only very recently been investigated. Although few details are known as yet, the data available indicate that one is also facing a complex situation involving the stress sub-proteome, cell viability, and metabolites such as glycerol, reducing sugars, and ethanol [24].

Whereas *S. cerevisiae*’s fermentative lifestyle is shared by other fermentative yeasts [25], its ethanol tolerance is only shared with other *Saccharomyces* yeasts [26]. A similar situation may be the case for CO_2_ pressure tolerance since *Saccharomyces* are the only known yeasts able to successfully complete the second fermentation of sparkling wine inside a hermetically closed glass bottle. Contrarily, the SO_2_ tolerance of *S. cerevisiae* is shared with other non-*Saccharomyces* fermentative yeasts. As strains of yeast are phenotypically diverse, the isolation and selection of *T. delbrueckii* strains resistant to any of the aforementioned stresses should be possible. Thereafter, genetic improvement through yeast breeding or the elimination of non-interesting alleles could be carried out. However, the lack of precise knowledge about the lifecycle of this yeast makes it difficult to design strategies for biotechnological improvement by using the classical genetic techniques already used for *S. cerevisiae* wine yeasts [27,28]. Besides this, theoretically, one could try to get *T. delbrueckii* to evolve similarly as *S. cerevisiae* did to improve ethanol, SO_2_, and CO_2_ pressure tolerance, as has been done by adaptive laboratory evolution to improve growth and ethanol production at high temperatures [13].

The present work describes a genetic study of four selected *T. delbrueckii* strains to eliminate possible recessive deleterious alleles in order to obtain new improved spore clones with enhanced fermentation capability. Then, we performed sequential isolations of spontaneous mutants resistant to different stressful conditions related to still and sparkling wine making. The main aim was to improve the overall fermentation performance of this yeast species to bring it as close as possible to that usually shown by *S. cerevisiae* wine yeasts. The utility of some of these improved mutants for commercial winery applications will be discussed.

## 2. Materials and Methods

### 2.1. Yeast Strains

*S. cerevisiae* (*Sc*) EX229 is a Klus-killer wine strain that kills other *S. cerevisiae* and *T. delbrueckii* yeasts [29]. *Sc* 85R4A is a non-killer, cycloheximide-resistant (cyh^R^) spore clone obtained from the *Sc* EX85R (originally named JP85R; [30]) wine yeast. These *S. cerevisiae* strains were used in this study as reference yeasts for still and sparkling wine fermentation. *T. delbrueckii* (*Td*) Kbarr EX1180 and *Td* EX1257 are prototrophic wine yeasts that kill all known types of *S. cerevisiae* killer and non-killer strains and non-killer *T. delbrueckii* strains. *Td* EX1180-11C4 and *Td* EX1257-CYH5 are cyh^R^ spontaneous mutants from *Td* EX1180 and *Td* EX1257, respectively. These strains had previously been selected for winemaking [9,10,12]. The genetic marker cyh^R^ allows easy traceability of the new mutants obtained from these yeasts. Industrial use of *Td* EX1180 and *Td* EX1257 is under patent application [31].

### 2.2. Culture Media and Phenotype Tests

Standard culture media were used for yeast growth [32]. YEPD broth contained 1% yeast extract, 2% peptone, and 2% glucose. YEPD agar is YEPD broth with 2% agar. YEPD+cyh is YEPD agar supplemented with 2 µg/mL cycloheximide [30]. YEPD+EtOH is YEP -agar supplemented with ethanol just before pouring the medium into Petri plates to a 5% or 10% (*v*/*v*) final concentration. SD agar contained 0.67% Yeast Nitrogen Base (without amino acids; with ammonium sulfate, Difco), 2% glucose and 2% Bacto-agar. SD+SO_2_ is SD agar buffered with 75 mM tartaric acid at pH 3.5, and supplemented with a freshly prepared 6% K_2_S_2_O_2_ stock solution that was added to each plate two hours before yeast seeding (125 or 250 mg/L SO_2_ final concentration) [28]. Macabeo grape must (21.3 °Brix, pH 3.2, malic acid 1.4 g/L, lactic acid 0.08 g/L) was sterilized by membrane filtration through a Millipore system (0.45 μm membrane). This sterile synthetic must was a modified version [33] of that described previously [34]. The sterile synthetic base wine used contained 1% yeast extract, 0.1% peptone, and 2.4% sucrose, 10% ethanol, 0.3% tartaric acid, and 0.2% malic acid, pH 3.1.

Standard procedures were used for the sporulation of yeast cultures and dissection of asci [35]. Yeast cells were grown on YEPD agar plates or in YEPD broth for two days at 30 °C and then transferred to sporulation plates (SPO; 1% potassium acetate, 0.1% yeast extract, 0.05% glucose, 2% agar) and incubated for 20 days at 25 °C. The percentage of sporulated yeasts was determined at this time. The tetrads (asci) from each yeast strain were dissected on YEPD plates using a micromanipulator, and then incubated for 5 days at 30 °C. Spore viability and spore clone size were determined at this time. Spore clones were tested for phenotype segregations by replica plating on agar plates for sugar fermentation (sucrose, maltose, galactose, melibiose, melezitose, trehalose, raffinose, starch and α-methyl glucoside), copper resistance, SO_2_ resistance, and H_2_S production as previously described [36]. Homothallism was determined by examining the ability of isolated spore clones to sporulate. Petite phenotype was analyzed on Yeast extract–Peptone–Glycerol medium (YPG) (1% yeast extract, 2% peptone, 3% glycerol, 2% agar, and 1% ethanol added after autoclaving).

### 2.3. Grape Must, Synthetic Must and Synthetic Base Wine Fermentations

Yeast cells were cultured in YEPD broth for 2 days at 30 °C, washed twice with sterile water, and inoculated into synthetic must or synthetic base wine (2–4 × 10 ^6^ cells/mL for *S. cerevisiae*, and 2–4 × 10^7^ cells/mL for *T. delbrueckii*). In parallel, a fermentation inoculated with *Sc* EX229 or *Sc* 85R4A was done as a reference positive control for each experiment. Fermentations were performed in 250 mL Erlenmeyer flasks with 60 mL of must or base wine, at 20 °C or 16–18 °C, respectively. Before base wine inoculation, when required, yeast cultures were adapted to growth in this medium as indicated below for sparkling wine making, using the different culture shaking procedures mentioned in the Results section. The density, °Brix, yeast growth (total and viable yeast cells), and dead cells were monitored. Cell death was determined by staining with methylene blue and the observation of yeast samples under a microscope with a Nomarski 60× objective. Since the morphological changes in the yeast cells during the second fermentation of sparkling wine was highly variable, the total amount of dead cells was calculated as the sum of blue, empty, and destroyed/autolyzed cells [12].

### 2.4. Base Wine Fermentation for Sparkling Wine Making

Cava-type sparkling wine was made using the traditional method in our experimental winery as previously described [12]. Two different commercial base wines were used, one from Garnacha red grapes (pH 3.20, 4.93 g/L total acidity, 0.87 g/L reducing sugars, 10.9% alcohol *v*/*v*) and another from Macabeo white grapes (pH 3.18, 5.7 g/L total acidity, 1.2 g/L reducing sugars, 10.8% alcohol *v*/*v*). Before base wine inoculation, each yeast culture was adapted to growth in each base wine as follows. Each yeast pellet from a 48-h YEPD broth culture was resuspended in sterile water supplemented with 2.4% sucrose and 0.2% diammonium phosphate ((NH4)_2_PO4) (2–4 × 10^9^ CFU/mL), and incubated at room temperature (18–22 °C) for about 2 h. This culture was then diluted with one volume of a mixture 1:1 water:base wine with 2.4% sucrose, and incubated for 5 h at 18–22 °C. Each culture was then diluted again with one volume of base wine with 2.4% sucrose and incubated overnight at room temperature. Finally, each culture was diluted again with 1.5 volumes of base wine with 2.4% sucrose, and incubated for 5 h at 18–22 °C. Occasional shaking of these cultures was done every 2–12 h as convenient. These yeast-adapted cultures contained a final cell concentration of 2–6 × 10^8^ CFU/mL. For sparkling wine making, base wine was supplemented with 2.4% sucrose and 0.02% diammonium phosphate, and single-inoculated with each adapted yeast culture in 0.75 L capped bottles in which high pressures above 6 atm could be reached after the second fermentation. Three replicates of each yeast’s second fermentation were done. The intended amount of yeast inoculum was 2–4 × 10^6^ viable cells/mL for *S. cerevisiae*, and 2–4 × 10^7^ viable cells/mL for *T. delbrueckii*. The second fermentation was done at 18–19 °C for the first 15 days, and thereafter at 12–14 °C. Samples for microbiological and chemical assay were taken at different times from 0 to 270 days. After 270 days of fermentation and aging, the sparkling wines were riddled for 30 days to move the lees to the bottle neck. Finally, after disgorging, aromatic compounds and organoleptic quality were assayed. A descriptive organoleptic analysis was done for each sparkling wine by an expert panel of 10 judges as previously described [9].

### 2.5. Determination of the Inoculated Yeast Proportion during Fermentation

The percentages of cyh^R^ genetically marked *Sc* 85R4A, *Td* EX1180-11C4, and *Td* EX1257-CYH5 yeasts were determined by replica-plating on YEPD+cyh [37]. The percentage of wild yeasts such as *Sc* EX229 was determined by mtDNA restriction pattern analysis [38]. When appropriate, this same procedure was used to validate the results obtained by replica-plating analysis.

### 2.6. Analytical Methods

Degrees Brix (°Brix) were measured using a digital refractometer. Alcohol content, pH, total acidity, volatile acidity, glucose and fructose, and density were determined using European Commission (EC) recommended methods [39]. Sparkling wine pressure was measured at room temperature using an aphrometer, and values were then corrected to 20 °C by using Henry’s law constant.

## 3. Results and Discussion

### 3.1. Genetic Characterization of T. delbrueckii: Sporulation Capability, Viability of Spores, Presence of Recessive Growth-Retarding Alleles, and Degree of Heterozygosity

**Figure 1:**
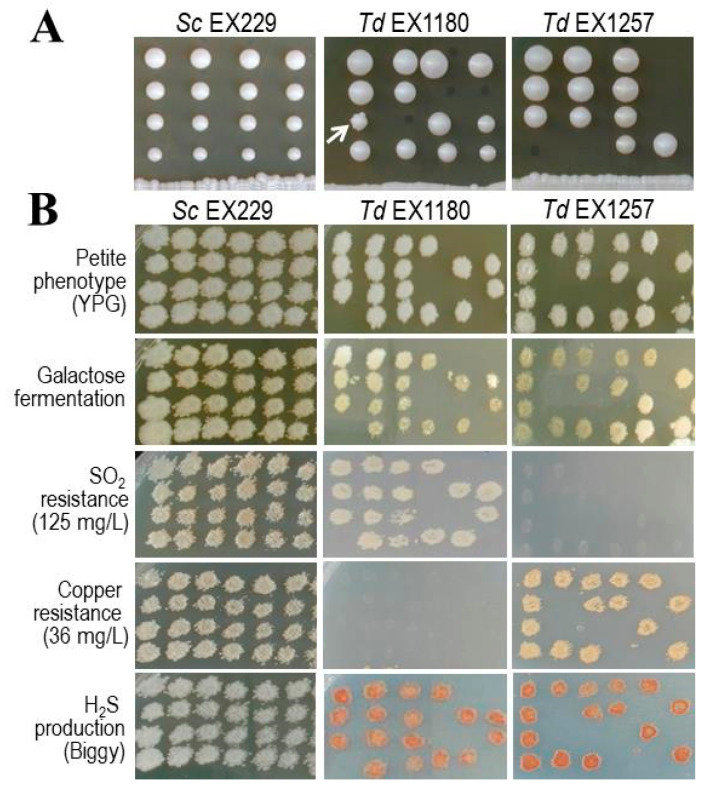
Genetic characterization of *T. delbrueckii*. (**A**) Spore colony size after tetrad dissection of *Sc* EX229, and *Td* EX1180 and *Td* EX1257 strains. Spore tetrads from separate asci are arranged along vertical lines. Sectored colony is indicated with an arrow. (**B**) Examples of some phenotype tests after replica plating of some spore clones on the media indicated (on the left). YPG, Yeast extract–Peptone–Glycerol medium.

The sporulation of *T. delbrueckii* in SPO medium was rather low (3.5–22% tetrads) compared to that of *S. cerevisiae* (55–74%). However, the viability of *T. delbrueckii* spores after tetrad dissection on YEPD agar was generally high (average 78%, 68%, 64%, and 69% for *Td* EX1180, *Td* EX1180-11C4, *Td* EX1257, and *Td* EX1257-CYH5, respectively), although not as high as that of *S. cerevisiae,* which most times was 100%. Most *T. delbrueckii* spore clones were large in size. Only two spore clones were medium to small in size and sectored, similar to those previously found for genetically unstable yeasts [28,40] (Figure 1A). Despite this, no pattern of genetic segregation for non-viable or small colonies bearing deleterious recessive alleles was found in *Td* EX1180. The phenotype of these two clones could therefore be due to some spontaneous mutation or metabolic deficiency that happened to affect some cells that then did not multiply or died, causing circular sectors of non-growth in the colony (i.e., no deleterious allele was detected that would be responsible for slow growth in rich medium, nor was a regular pattern for genetic segregation of non-viable spores found). If any deleterious or lethal alleles existed in any of these *T. delbrueckii* strains, they could be associated with spores that did not germinate. In view of these results, it does not seem reasonable to try to select spore clones free of recessive growth-retarding alleles as a basic, and much less exclusive, strategy to address the genetic improvement of these *T. delbrueckii* strains, contrary to the case previously described for *S. cerevisiae* wine strains [28].

All *T. delbrueckii* strains showed homozygosity for most phenotypes analyzed (fermentation of sucrose, maltose, galactose, melibiose, melezitose, trehalose, raffinose, starch, and α-methyl glucoside; copper resistance; H_2_S production; homothallism; and petite phenotype) except for resistance to 125 mg/L SO_2_, for which differences between the spore clones of some tetrads were sometimes observed. Nonetheless, no segregation pattern was observed for any of the phenotypes analyzed. *T. delbrueckii* strains and their spore clones were resistant to 5% ethanol but not 10%. *Td* EX1257, *Td* EX1257-CYH5, and their spore clones did not resist 125 mg/L SO_2_. *Td* EX1180, *Td* EX1180-11C4, and their spore clones were resistant to 125 mg/L SO_2_ but not to 250 mg/L. All *T. delbrueckii* strains and their spore clones produced more H_2_S (brown color on Biggy agar) than *S. cerevisiae* (beige to light brown). *Td* EX1257, *Td* EX1257-CYH5, and their spore clones were resistant to 36 mg/L copper but not *Td* EX1180, *Td* EX1180-11C4, or their spore clones (Figure 1B). This high degree of homozygosity for the phenotypes analyzed makes us suspect that our *T. delbrueckii* strains are haploid yeasts that mate among themselves or with their daughter buds to originate diploid cells immediately before undergoing meiosis to generate tetrads. Indeed, conjugative tubes were observed in all these yeasts before obtaining spores in SPO medium. This circumstance rules out any possibility of detecting heterozygous loci, unless two strains with different alleles for the analyzed genes mated immediately before sporulation.

### 3.2. Fermentation Capability of T. delbrueckii Spore Clones

**Figure 2:**
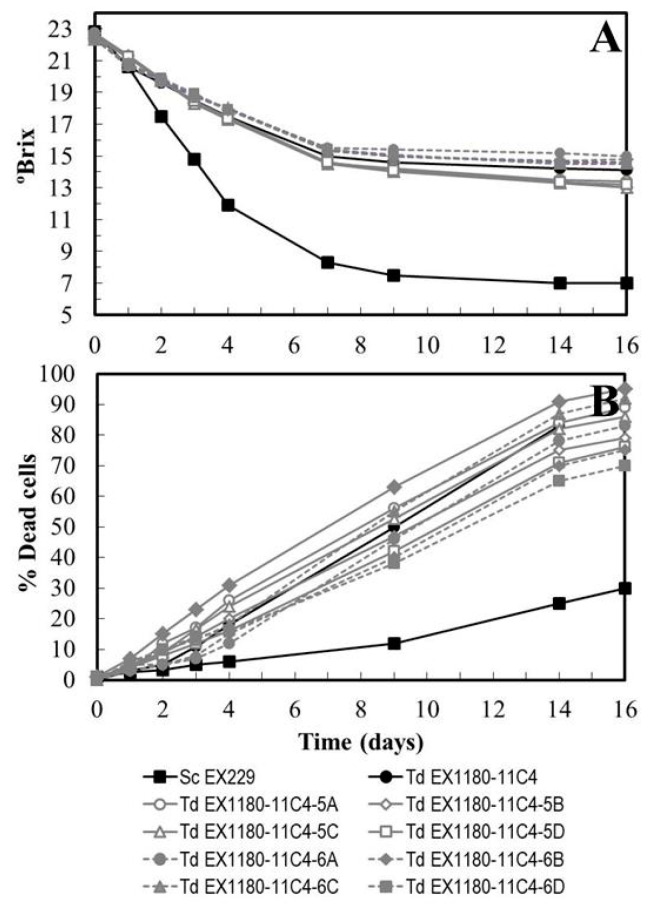
Synthetic must fermentation kinetics of eight large colony size spore clones from two complete tetrads of *Td* EX1180-11C4 (5A, 5B, 5C, and 5D; and 6A, 6B, 6C, and 6D). (**A**) Degrees Brix (°Brix) of must/wine. (**B**) Percentage of dead cells. Data are the mean values of three fermentations inoculated with each yeast strain. Standard deviations were less than 8% of the means. The degree of dominance throughout fermentation of each inoculated yeast strain was 100%.

To avoid any uncertainty and totally rule out the possible influence of any deleterious allele related to the growth of *T. delbrueckii* that we had not detected, eight large colony-size spore clones from two tetrads of *Td* EX1180-11C4 (5A, 5B, 5C, and 5D; and 6A, 6B, 6C, and 6D) and another four spore clones from a *Td* EX1180 tetrad (5A, 5B, 5C, and 5D) were chosen to perform fermentation of synthetic must. All spore clones had slower fermentation kinetics than *S. cerevisiae*, and were similar to the corresponding parental strain (see Figure 2A for those from *Td* EX1180-11C4). The increase in cell death during the fermentation of *T. delbrueckii* spore clones was still greater than that of *S. cerevisiae* in all cases, and not very different from that of the parental *T. delbrueckii* strain (Figure 2B). However, to ensure that the new improved yeasts did not contain any deleterious alleles related to growth, the new spontaneous mutants of *T. delbrueckii* were subsequently isolated from some of these well-growing spore clones (see below). This may be important because the presence of any undetected deleterious allele in the parental yeast could cause some fermentation problems in environmental conditions different from those in the present study.

### 3.3. Isolation and Characterization of New T. delbrueckii Mutants Resistant to SO_2_ and Ethanol

**Figure 3:**
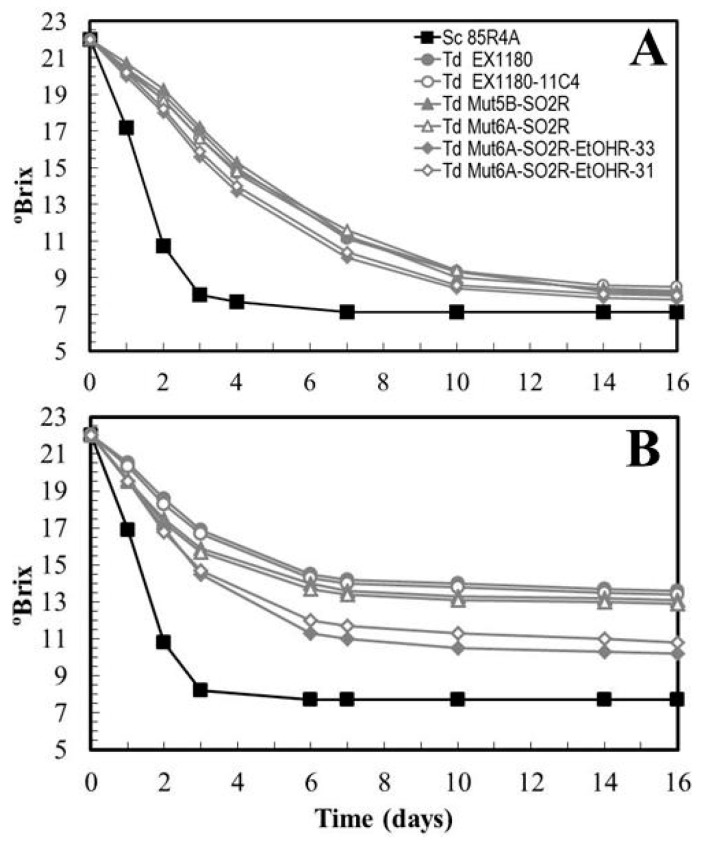
Fermentation kinetics of *T. delbrueckii* SO_2_ resistant mutants (*Td* Mut5B-SO2R and *Td* Mut6A-SO2R) and SO_2_ + ethanol resistant mutants (*Td* Mut6A-SO2R-EtOHR-31 and *Td* Mut6A-SO2R-EtOHR-33) in synthetic must (**A**) and synthetic must containing 50 mg/L SO_2_ (**B**). Data are the mean values of three fermentations inoculated with each yeast strain. Standard deviations were less than 10% of the means. The degree of dominance throughout fermentation of each inoculated yeast strain was 100%.

Several *Td* EX1180-11C4 (27) and *Td* EX1257-CYH5 (18) spore clones were plated onto YEPD plates supplemented with 250 mg/L SO_2_. Resistant papillae were isolated only from the *Td* EX1180-11C4-5B and -6A spore clones. No papilla were isolated from the *Td* EX1257-CYH5 spore clones. A purified colony was selected from *Td* EX1180-11C4-5B and -6A papillae: *Td* Mut5B-SO2R and *Td* Mut6A-SO2R, respectively. Subsequently, we were able to isolate new spontaneous mutants capable of growing on YEPD plates with 10% ethanol, but only from Mut6A-SO2R. The authenticity of these new *T. delbrueckii* mutants was verified by analyzing the morphology of their vegetative cells, spores, killer phenotype, cycloheximide resistance, viral dsRNA profile, and mtDNA Restriction Fragment Length Polymorphism (RFLP) profile. The results of all tests agreed with those corresponding to the parental strain *Td* EX1180-11C4 for all the mutants (i.e., one can rule out that these mutants might have come from other contaminating yeasts). The fermentation capability in synthetic must of the SO_2_ resistant mutants *Td* Mut5B-SO2R and *Td* Mut6A-SO2R was similar to that of their parental yeast *Td* EX1180-11C4. However, a slight improvement was seen in the SO_2_ + ethanol resistant mutants (named *Td* Mut6A-SO2R-EtOHR-31 and *Td* Mut6A-SO2R-EtOHR-33), although this improvement became irrelevant after 14 days of fermentation (Figure 3A). The fermentation capability of *Td* Mut5B-SO2R and Mut6A-SO2R in synthetic must supplemented with 50 mg/L SO_2_ was also slightly better than that of the parental strain during the first days of fermentation, but this improvement also became irrelevant after the sixth day of fermentation, when approximately 5% ethanol was reached. However, an evident and relevant improvement was observed in *Td* Mut6A-SO2R-EtOHR-31 and *Td* Mut6A-SO2R-EtOHR-33 that was maintained throughout fermentation (Figure 3B). These results indicate that in must fermentation, where there is a rapid increase in ethanol concentration, the possible fermentative improvement that the resistance to SO_2_ phenotype would provide to *T. delbrueckii* is only relevant if the yeast strain also has increased ethanol resistance.

### 3.4. Isolation and Fermentation Capability of New T. delbrueckii Mutants Resistant to High CO_2_ Pressure (HP^R^) from Mutants already Resistant to SO_2_ and Ethanol

**Figure 4:**
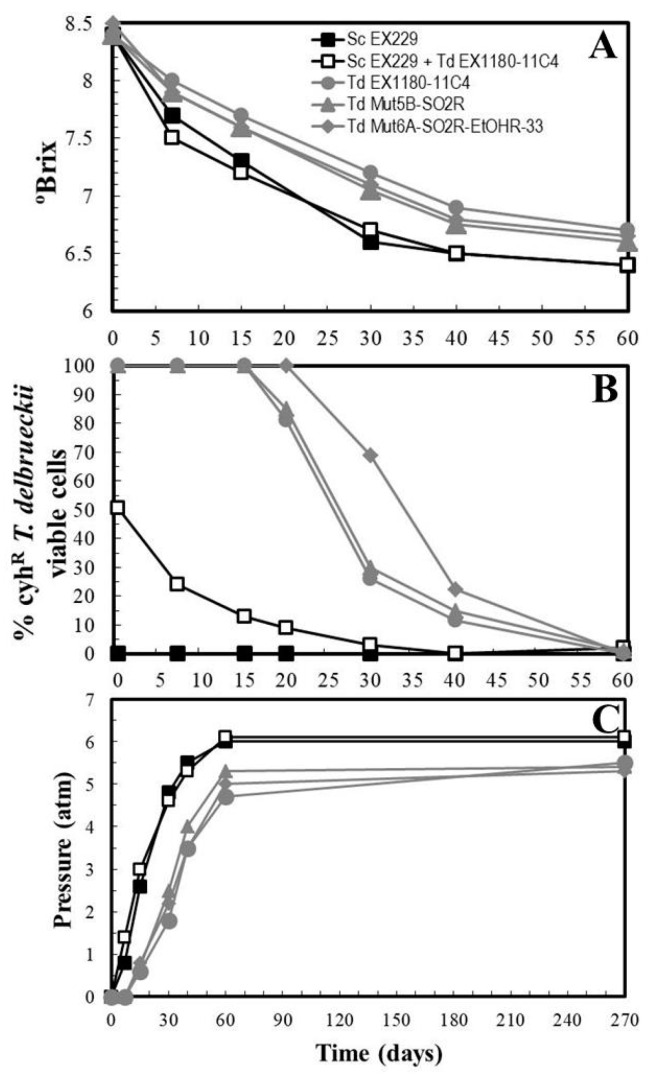
Fermentation kinetics and yeast-population dynamics during sparkling wine second-fermentations inoculated with *T. delbrueckii* mutants resistant to SO_2_ and ethanol. Garnacha base wine was single inoculated with *Sc* EX229, *Td* EX1180-11C4, *Td* Mut5B-SO2R or *Td* Mut6A-SO2R-EtOHR-33, or mixed inoculated with *Sc* EX229 + *Td* EX1180-11C4. (**A**) Evolution of sugar consumption (°Brix). (**B**) Percentage of cyh^R^ yeast cells in each fermentation. Note that the cyh^R^ *T. delbrueckii* viable cells tended to disappear as CO_2_ pressure increased. (**C**) Pressure inside the bottle. Data are the mean values of three fermentations inoculated with each yeast strain. Standard deviations were less than 13% of the means.

One mutant of each type was selected to make rosé sparkling wine (cava) under cellar conditions: *Td* Mut5B-SO2R (resistant to SO_2_) and *Td* Mut6A-SO2R-EtOHR-33 (resistant to SO_2_ and ethanol). As was the case with the parental strain *Td* EX1180-11C4, no *T. delbrueckii* mutant was able to dominate the entire process to the end and complete the second in-bottle fermentation, while this was accomplished successfully by the reference yeast *Sc* EX229. Fermentation was also successfully accomplished by a mixed inoculum of *Sc* EX229 + *Td* EX1180-11C4, but in this case, the *T. delbrueckii* yeasts were quickly overcome by *S. cerevisiae* yeasts from the beginning of fermentation. All single-inoculated *T. delbrueckii* yeasts began to die and were overcome by contaminant *Saccharomyces* yeasts after 20 days of fermentation, when a CO_2_ pressure of about 1 atm was reached. Contaminant yeasts are common in industrial base wine, and here were responsible for the completion of fermentations that had been single inoculated with *T. delbrueckii*. However, in terms of fermentation kinetics and yeast survival together, *Td* Mut6A-SO2R-EtOHR-33 was better than the parental strain and *Td* Mut5B-SO2R during the first 40 days of fermentation (Figure 4A,B). The sparkling wines inoculated with the mutants were slightly fruitier, had less aging notes, and greater amounts of some ethyl esters than those inoculated with their parental yeast. However, the relevance of these results is far from clear since how much of this effect was due to the involvement of contaminant *Saccharomyces* yeasts cannot be specified.

Yeast colonies were isolated on YEPD agar inoculated with samples from the sparkling wines that were single inoculated with *T. delbrueckii* yeasts taken at 30, 40, and 60 days of fermentation. Those colonies that morphologically seemed to correspond to this species were pre-selected, and those that seemed to be *S. cerevisiae* were discarded. After 60 days, when 4.5 atm pressure had been surpassed (Figure 4C), no viable *T. delbrueckii* yeasts were isolated. A total of 40 *T. delbrueckii* colonies were pre-screened to try to obtain possible spontaneous mutants resistant to high CO_2_ pressure (HP^R^) that had survived as long as possible during the second in-bottle fermentation (40 days of fermentation). The identity of these possible HP^R^ mutants was verified by analysis of cell morphology, killer phenotype, resistance to cycloheximide, presence of viral dsRNA, RFLPs of mtDNA, and sequencing of Internal Transcribed Spacer of ribosomal DNA (ITS). Subsequently, this pre-selection was restricted to eighteen HP^R^ mutants: ten from the sparkling wine inoculated with the parental strain *Td* EX1180-11C4, two from that inoculated with *Td* Mut5B-SO2R, and six from that inoculated with Td Mut6A-SO2R-EtOHR-33, with full certainty that these mutants came from the corresponding parental *T. delbrueckii* and not from any other yeast contaminant.

**Figure 5:**
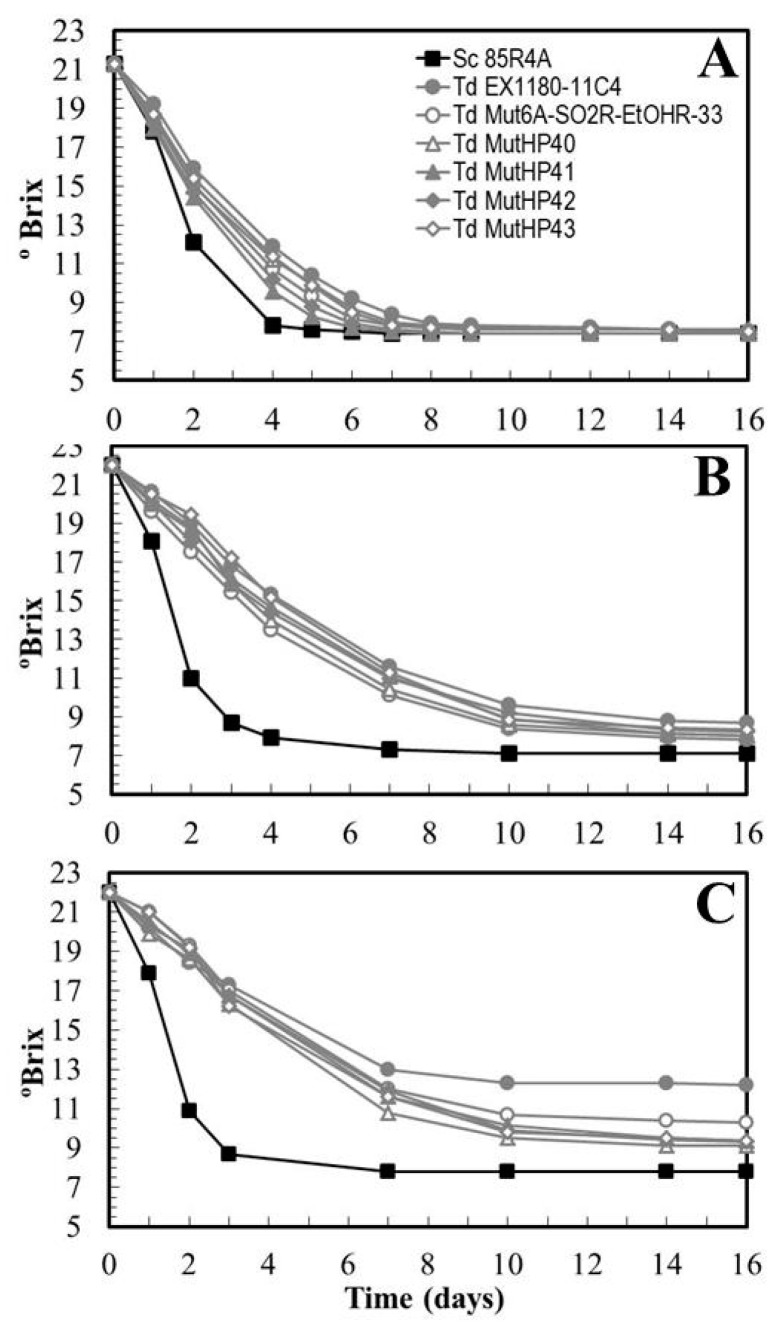
Fermentation kinetics of some *T. delbrueckii* high CO_2_ pressure resistant (HP^R^) mutants inoculated in sterile fresh grape must (**A**), synthetic must (**B**), and synthetic must supplemented with 100 mg/L SO_2_ (**C**). Data are the mean values of three fermentations inoculated with each yeast strain. Standard deviations were less than 11% of the means. The degree of dominance throughout fermentation of each inoculated yeast strain was 100%.

These eighteen HP^R^ mutants were inoculated into Macabeo grape must, synthetic must, and synthetic must with 100 mg/L SO_2_. Fermentative vigor and the ability to complete fermentation were analyzed. Some improvement was observed for some HP^R^ mutants (such as *Td* MutHP41 and *Td* MutHP42) with respect to their parents in fresh grape must fermentations (Figure 5A), but this improvement was less clear in synthetic must (Figure 5B). Fermentation kinetics were in most cases faster in fresh grape must than in synthetic must, and were even slower when 100 mg/L SO_2_ had been added. This was especially evident for the *Td* EX1180-11C4 yeast, which is the most sensitive to SO_2_ and ethanol. The HP^R^ mutants had faster fermentation kinetics than their parental yeast *Td* Mut6A-SO2R-EtOHR-33, being less affected by the presence of SO_2_. Unfortunately, no mutant completed this type of fermentation within 16 days, which is more than twice the time (seven days) required by the *Sc* 85R4A reference yeast to complete fermentation (Figure 5C). All these results indicate that the synthetic must fermentation conditions were so severe that SO_2_ resistance of the mutants was not a definitive advantage for the completion of fermentation. Once again, it seems that the high and rapid increase of ethanol concentration in this type of fermentation was the limiting factor for maintaining the viability and fermentation rate of *T. delbrueckii* yeasts, even though the new mutants were resistant to SO_2_ and this compound was present, or even that they were more resistant to ethanol and CO_2_ pressure than their parental yeast.

**Figure 6:**
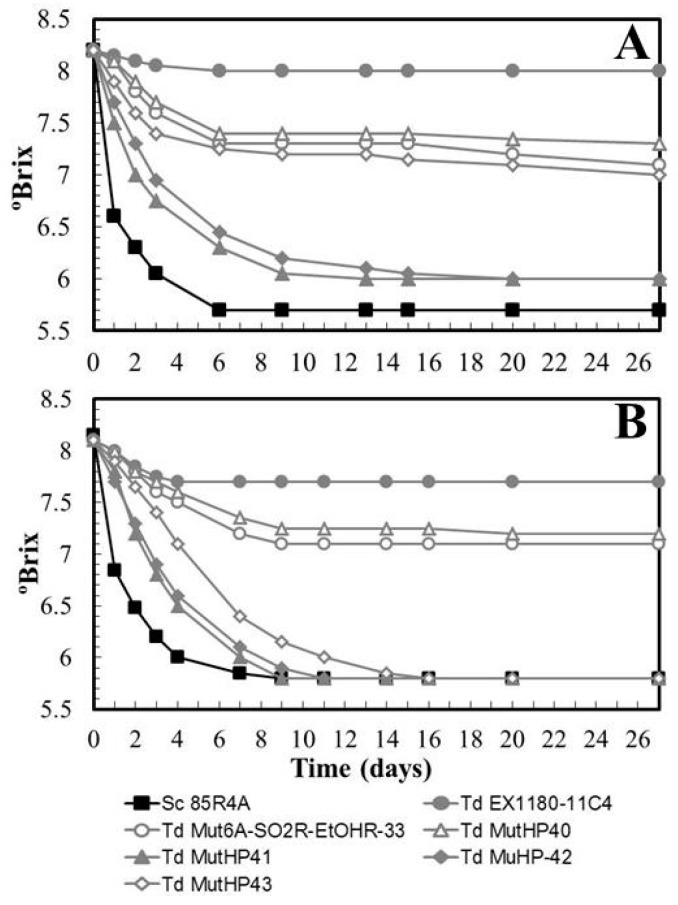
Fermentation kinetics of some *T. delbrueckii* HP^R^ mutants inoculated in synthetic base wine supplemented with 50 mg/L (**A**) or 30 mg/L SO_2_ (**B**). Data are the mean values of three fermentations inoculated with each yeast strain. Standard deviations were less than 8% of the means. The degree of dominance throughout fermentation of each inoculated yeast strain was 100%.

However, most HP^R^ mutants showed a relevant improvement in synthetic base wine fermentations supplemented with 50 mg/L SO_2_ (Figure 6A). Furthermore, they were able to complete the fermentation when the amount of SO_2_ was reduced to 30 mg/L just 3–4 days after the reference yeast *Sc* 85R4A. Only one of the selected mutants, MutHP40, did not improve with respect to its direct parental strain *Td* Mut6A-SO2R-EtOHR-33 (Figure 6B). This SO_2_ concentration is similar to that commonly used in the cava-type sparkling-wine industry (between 15 and 25 mg/L). Therefore, the two mutants with the best fermentation kinetics, *Td* MutHP41 and *Td* MutHP42, were selected because, in the presence of 30–50 mg/L of SO_2_, they showed great improvements over their original parental yeast (*Td* EX1180-11C4) and the intermediate mutant (*Td* Mut6A-SO2R-EtOHR-33) from which they directly proceeded, and also because they were the mutants whose fermentation kinetics were closest to that of the reference yeast *Sc* 85R4A. Considering the number (18) of initially pre-selected HP^R^ clones, one can estimate an 11% success rate in our strategy to obtain new improved strains of *T. delbrueckii* for base wine fermentation. However, only some HP^R^ mutants improved the capability for synthetic must fermentation compared to their parental yeast, and this improvement was of little relevance because they did not complete the fermentation in a time that is reasonable for the commercial production of still wines. As noted above, this may be because the increase of ethanol concentration during must fermentation is much greater and faster than in sparkling wine fermentations. In other words, a different, more specific strategy from that used in this present work is probably required to improve the efficiency of *T. delbrueckii* yeasts for still wine fermentation. It was not enough to select mutants resistant to SO_2_ and ethanol in supplemented culture plates, or HP^R^ mutants capable of better resisting the CO_2_ pressure during the slow second fermentation of traditional sparkling wine.

**Figure 7:**
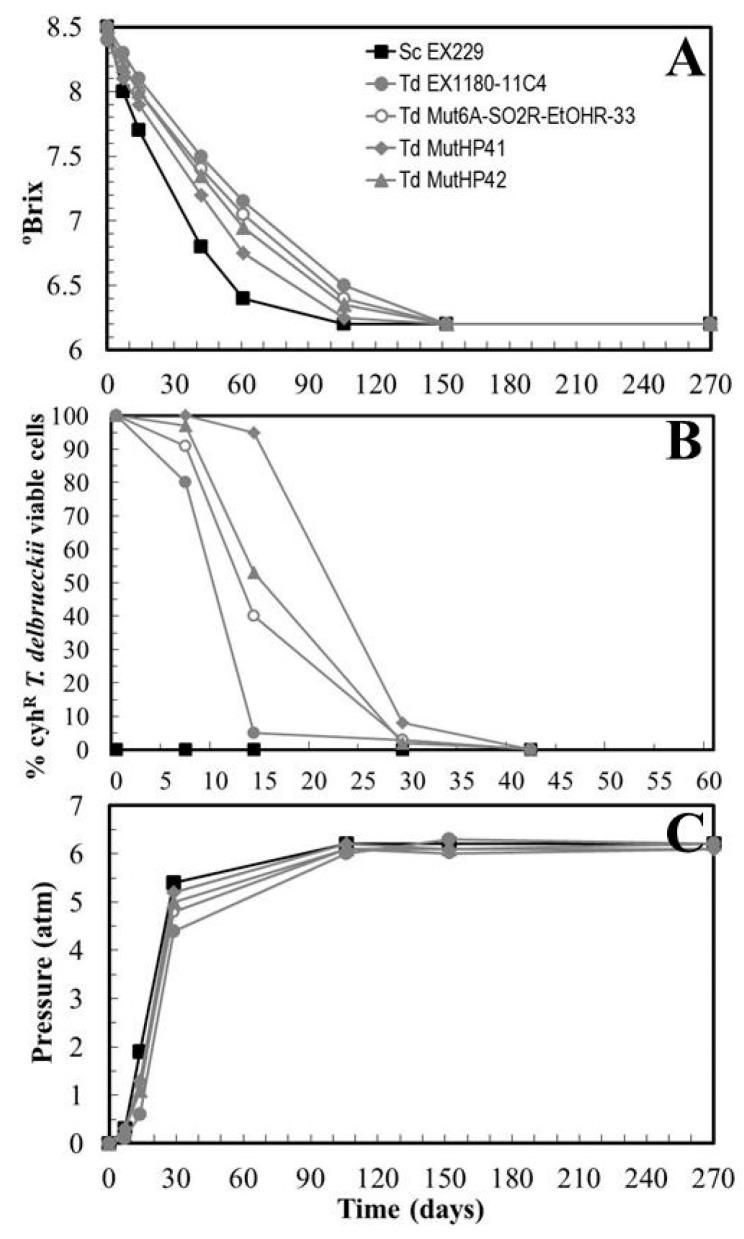
Fermentation kinetics and yeast population dynamics during sparkling wine second-fermentations inoculated with selected *T. delbrueckii* HP^R^ mutants. Macabeo base wine was single inoculated with *Sc* EX229, *Td* Mut6A-SO2R-EtOHR-33, *Td* MutHP41, or *Td* MutHP42. (**A**) Evolution of sugar consumption (Degrees Brix). (**B**) Percentage of cyh^R^ yeast cells in each fermentation. (**C**) Pressure inside the bottle. Data are the mean values of three fermentations inoculated with each yeast strain. Standard deviations were less than 15% of the means.

New sparkling wines were made with the two HP^R^ selected mutants, *Td* MutHP41 and *Td* MutHP42, to repeat the previous strategy for selecting new yeast clones that were even more resistant to high CO_2_ pressure. Once again, neither of the two inoculated mutants was able to dominate and complete fermentation inside the glass bottle. After day 30, when the pressure reached was greater than 4 atm, the *T. delbrueckii* yeasts were overwhelmed by the *Saccharomyces* yeasts contaminating the industrial base wine. This time however, the two mutants had better fermentation kinetics and dominance than their parental yeast. *Td* MutHP41 was clearly the best of the *T. delbrueckii* yeasts (Figure 7A–C). These results confirm that *Td* MutHP41 is really an improvement for industrial base wine fermentation over its parental yeast. However, neither of the two selected mutants achieved the fermentation speed of the *S. cerevisiae* reference yeast under these cellar conditions. That is, although *Td* MutHP41 might be able to slowly complete second sparkling wine fermentation, it could well be overwhelmed by contaminating *Saccharomyces* yeasts that can complete fermentation faster than this *T. delbrueckii* mutant. In this situation, one would always have some uncertainty regarding the final organoleptic quality of commercially produced wines. It has to be borne in mind that wine quality should be equal or very similar in all the bottles of sparkling wine made with the same raw material and under the same cellar conditions.

We re-isolated new colonies and pre-selected 24 new putative HP^R^ clones from the Macabeo sparkling wine samples at the maximum pressure points where viable *T. delbrueckii* yeasts still remained (14 and 29 days of fermentation). After the appropriate cellular and molecular analyses, 13 clones (five from *Td* MutHP41 and eight from *Td* MutHP42) were selected for the fermentation of synthetic wine supplemented with 30 mg/L SO_2_. Unfortunately, none of the new preselected clones improved the fermentation kinetics of the parental *Td* MutHP41 and *Td* MutHP42. This indicates that repeating the isolation and selection procedure to obtain new reinforced HP^R^ mutants from previously selected HP^R^ mutants was not a sound strategy to continue improving the fermentative capability of *T. delbrueckii* under high CO_2_ pressure.

### 3.5. Improving the Fermentation Capability of HP^R^ Mutants by Conditioning Yeast Culture before Base Wine Inoculation

**Figure 8:**
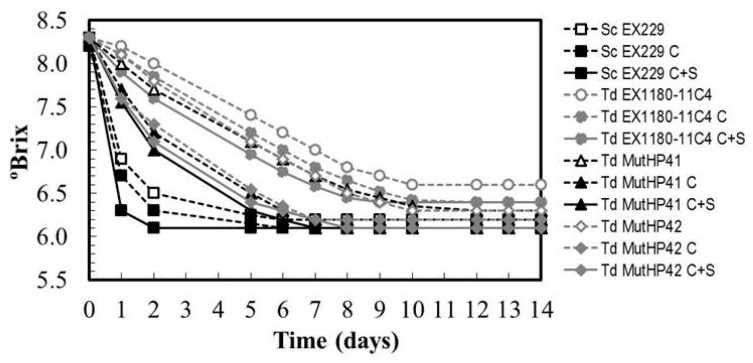
Fermentation kinetics of some *T. delbrueckii* HP^R^ mutants inoculated in synthetic base wine supplemented with 30 mg/L SO_2_. Before inoculation, the yeast cultures were unconditioned (no indication following the name of the strain), conditioned with occasional shaking (C), or conditioned with continuous shaking (C+S). Data are the mean values of three fermentations inoculated with each yeast strain. Standard deviations were less than 9% of the means. The degree of dominance throughout fermentation of each inoculated yeast strain was 100%.

Since *T. delbrueckii* has a higher oxygen requirement to grow than *S. cerevisiae*, we tested two protocols to condition the yeast cultures prior to inoculation of base wine (30 mg/L SO_2_): one with occasional shaking every 4–12 h (as is usually done to make commercial sparkling wine), and another with continuous shaking. In this way, one can evaluate how the oxygen supply during the adaptation of these yeasts to the base wine environment improves their resistance to ethanol, and hence their fermentation capability. Occasional shaking improved the fermentation capability of all the yeasts, including the *S. cerevisiae* reference strain which already had excellent fermentation capability even without previous culture conditioning. The most relevant improvements were again found for *Td* MutHP41 and *Td* MutHP42. Continuous shaking further improved the fermentative capability of all yeasts, and especially that of *T. delbrueckii* yeasts. Interestingly, *Td* MutHP42 improved its fermentation capability to values close to *S. cerevisiae* after seven days of fermentation (i.e., once the fermentative capability of the new *Td* MutHP41 and *Td* MutHP42 mutants had been improved with respect to the parental *T. delbrueckii* yeast, it was still possible to further improve their base wine fermentation efficacy by prior conditioning with continuous shaking to provide extra oxygen to the yeast culture) (Figure 8). However, since the fermentation capability of these mutants was still less than that of *S. cerevisiae*, involvement of this latter yeast may still be necessary to ensure that the second fermentation of sparkling wine is completed in a reasonable time for commercial wineries, preferably within two months. The novelty when using these new HP^R^ mutants, conditioned with continuous oxygen supply, is that they would stay alive longer than their parental yeast during second fermentation inside the bottle, thus giving the wine more of the organoleptic characteristics of *T. delbrueckii*. Furthermore, this is particularly interesting in reducing the inoculum size of these yeasts to the level normally used for *S. cerevisiae* (from 1–2 × 10^7^ to 1–2 × 10^6^ CFU/mL. This would mean lower economic costs and could prevent the appearance of odors related to the production of H_2_S due to the accumulation of excess amounts of dead yeasts.

### 3.6. Genetic Stability of HP^R^ Mutants

**Figure 9:**
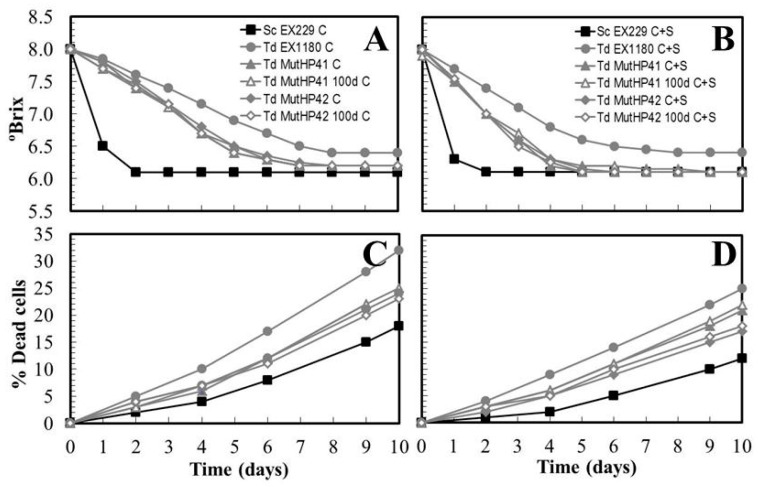
Genetic stability of HP^R^ mutants. Fermentation kinetics of synthetic base wine supplemented with 30 mg/L SO_2_ and inoculated with the mutants before and after 100 doublings (100d) on YEPD agar. Before inoculation, yeast cultures were conditioned with occasional shaking (C) or continuous shaking (C+S). Data are the mean values of three fermentations inoculated with each yeast strain. Standard deviations were less than 14% of the means. The degree of dominance throughout fermentation of each inoculated yeast strain was 100%. (**A**,**C**) Yeast cultures conditioned with occasional shaking. (**B**,**D**) Yeast cultures conditioned with continuous shaking.

Genetic stability of the new HP^R^ mutants after 100 doublings in rich non-selective culture medium (YEPD) was analyzed as previously described [41]. All the mutants maintained the genetic markers after 100 doublings. They also maintained their fermentative capability (Figure 9A,B) and the ability to remain viable longer than their parental strain (Figure 9C,D) throughout the fermentation of synthetic base wine, regardless of the type of culture conditioning protocol used (Figure 9A,C, vs. Figure 9B,D). This indicates that *Td* MutHP41 and *Td* MutHP42 are genetically stable enough to be considered for production at the industrial scale and marketed for use in industrial cellars, with no apparent risk that they might easily lose their new biotechnological properties. We are currently preparing to test these new mutants in commercial cellars for several consecutive years to validate their capability to improve sparkling wine quality, or to make differentiated alternative wines.

## 4. Conclusions

No deleterious recessive alleles related to cell growth were found in the *T. delbrueckii* yeasts analyzed, probably because they are haploid strains in their vegetative phase, in which this type of allele would tend to disappear. Consequently, the elimination of alleles of this type cannot be used as a main strategy for the genetic improvement of these non-*Saccharomyces* yeasts. Isolation of spontaneous mutants resistant to SO_2_ and ethanol seems to be a good strategy to slightly improve the fermentative efficiency of *T. delbrueckii* in must and base wine. Sequential isolation of HP^R^ mutants from previously obtained mutants resistant to SO_2_ and ethanol was required to obtain new mutants with significantly improved efficacy for the second fermentation of sparkling wine. These new mutants were genetically stable enough to be considered for industrial production, and their fermentative capability was further improved by continuously supplying oxygen during the conditioning stage before yeast culture inoculation in base wine.

## References

[1] Benito S. (2018). The impact of *Torulaspora delbrueckii* yeast in winemaking. Appl. Microbiol. Biotechnol..

[2] Ramírez M., Velázquez R. (2018). The yeast *Torulaspora delbrueckii*: An interesting but difficult-to-use tool for winemaking. Fermentation.

[3] Balmaseda A., Bordons A., Reguant C., Bautista-Gallego J. (2018). Non-*Saccharomyces* in wine: Effect upon *Oenococcus oeni* and malolactic fermentation. Front. Microbiol..

[4] Mauricio J.C., Millán C., Ortega J.M. (1998). Influence of oxygen on the biosynthesis of cellular fatty acids, sterols and phospholipids during alcoholic fermentation by *Saccharomyces cerevisiae* and *Torulaspora delbrueckii*. World J. Microbiol. Biotechnol..

[5] Visser W., Scheffers W.A., Batenburg-van der Vegte W.H., van Dijken J.P. (1990). Oxygen requirements of yeasts. Appl. Environ. Microbiol..

[6] Hanl L., Sommer P., Arneborg N. (2005). The effect of decreasing oxygen feed rates on growth and metabolism of *Torulaspora delbrueckii*. Appl. Microbiol. Biotechnol..

[7] González-Royo E., Pascual O., Kontoudakis N., Esteruelas M., Esteve-Zarzoso B., Mas A., Canals J.M., Zamora F. (2015). Oenological consequences of sequential inoculation with non-*Saccharomyces* yeasts (*Torulaspora delbrueckii* or *Metschnikowia pulcherrima*) and *Saccharomyces cerevisiae* in base wine for sparkling wine production. Eur. Food Res. Technol..

[8] García M., Greetham D., Wimalasena T.T., Phister T.G., Cabellos J.M., Arroyo T. (2016). The phenotypic characterization of yeast strains to stresses inherent to wine fermentation in warm climates. J. Appl. Microbiol..

[9] Velázquez R., Zamora E., Alvarez M.L., Hernández L.M., Ramírez M. (2015). Effects of new *Torulaspora delbrueckii* killer yeasts on the must fermentation kinetics and aroma compounds of white table wine. Front. Microbiol..

[10] Ramírez M., Velázquez R., Maqueda M., Zamora E., López-Piñeiro A., Hernández L.M. (2016). Influence of the dominance of must fermentation by *Torulaspora delbrueckii* on the malolactic fermentation and organoleptic quality of red table wine. Int. J. Food Microbiol..

[11] García-Ríos E., Guillamón J.M. (2019). Sulfur dioxide resistance in *Saccharomyces cerevisiae*: Beyond SSU1. Microb. Cell.

[12] Velázquez R., Zamora E., Álvarez M.L., Ramírez M. (2019). Using *Torulaspora delbrueckii* killer yeasts in the elaboration of base wine and traditional sparkling wine. Int. J. Food Microbiol..

[13] Caspeta L., Chen Y., Ghiaci P., Feizi A., Buskov S., Hallström B.M., Petranovic D., Nielsen J. (2014). Altered sterol composition renders yeast thermotolerant. Science.

[14] Lam F.H., Ghaderi A., Fink G.R., Stephanopoulos G. (2014). Engineering alcohol tolerance in yeast. Science.

[15] Caspeta L., Castillo T., Nielsen J. (2015). Modifying yeast tolerance to inhibitory conditions of ethanol production processes. Front. Bioeng. Biotechnol..

[16] Chen H., Jin S. (2006). Effect of ethanol and yeast on cellulase activity and hydrolysis of crystalline cellulose. Enzym. Microb. Technol..

[17] Casey G.P., Ingledew W.M.M. (1986). Ethanol tolerance in yeasts. CRC Crit. Rev. Microbiol..

[18] Piper P.W. (1995). The heat shock and ethanol stress responses of yeast exhibit extensive similarity and functional overlap. FEMS Microbiol. Lett..

[19] D’Amore T., Panchal C.J., Russell I., Stewart G.G. (1989). A study of ethanol tolerance in yeast. Crit. Rev. Biotechnol..

[20] Divol B., du Toit M., Duckitt E. (2012). Surviving in the presence of sulphur dioxide: Strategies developed by wine yeasts. Appl. Microbiol. Biotechnol..

[21] García-Ríos E., Nuévalos M., Barrio E., Puig S., Guillamón J.M. (2019). A new chromosomal rearrangement improves the adaptation of wine yeasts to sulfite. Environ. Microbiol..

[22] Yuasa N., Nakagawa Y., Hayakawa M., Iimura Y. (2004). Distribution of the sulfite resistance gene SSU1-R and the variation in its promoter region in wine yeasts. J. Biosci. Bioeng..

[23] Zimmer A., Durand C., Loira N., Durrens P., Sherman D.J., Marullo P. (2014). QTL dissection of Lag phase in wine fermentation reveals a new translocation responsible for *Saccharomyces cerevisiae* adaptation to sulfite. PLoS ONE.

[24] Porras-Agüera J.A., Román-Camacho J.J., Moreno-García J., Mauricio J.C., Moreno J., García-Martínez T. (2020). Effect of endogenous CO_2_ overpressure on the yeast “stressome” during the “prise de mousse” of sparkling wine. Food Microbiol..

[25] Hagman A., Säll T., Compagno C., Piskur J. (2013). Yeast “make-accumulate-consume” life strategy evolved as a multi-step process that predates the whole genome duplication. PLoS ONE.

[26] Williams K.M., Liu P., Fay J.C. (2015). Evolution of ecological dominance of yeast species in high-sugar environments. Evolution.

[27] Ramírez M., Pérez F., Regodón J.A. (1998). A simple and reliable method for hybridization of homothallic wine strains of *Saccharomyces cerevisiae*. Appl. Environ. Microbiol..

[28] Ramírez M., Regodon J.A., Pérez F., Rebollo J.E. (1999). Wine yeast fermentation vigor may be improved by elimination of recessive growth-retarding alleles. Biotechnol. Bioeng..

[29] Rodríguez-Cousiño N., Maqueda M., Ambrona J., Zamora E., Esteban E., Ramírez M. (2011). A new wine *Saccharomyces cerevisiae* double-stranded RNA virus encoded killer toxin (Klus) with broad antifungal activity is evolutionarily related to a chromosomal host gene. Appl. Environ. Microbiol..

[30] Pérez F., Regodón J.A., Valdés M.E., De Miguel C., Ramírez M. (2000). Cycloheximide resistance as marker for monitoring yeasts in wine fermentations. Food Microbiol..

[31] Ramírez M., Velázquez R., Maqueda M., López-Piñeiro A., Ribas J.C. (2015). A new wine *Torulaspora delbrueckii* killer strain with broad antifungal activity and its toxin-encoding double-stranded RNA virus. Front. Microbiol..

[32] Guthrie C., Fink G.R. (1991). Guide to yeast genetics and molecular biology. Methods Enzymol..

[33] OIV (2012). Guidelines for the Characterization of Wine Yeasts of the Genus Saccharomyces Isolated from Vitivinicultural Environments.

[34] Henschek P.A., Jiranek V., Fleet G.H. (1993). Yeast metabolism of nitrogen compounds. Wine Microbiology and Biotechnology.

[35] Kaiser C., Michaelis S., Mitchell A. (1994). Methods in Yeast Genetics.

[36] Mortimer R.K., Romano P., Suzzi G., Polsinelli M. (1994). Genome renewal: A new phenomenon revealed from a genetic study of 43 strains of *Saccharomyces cerevisiae* derived from natural fermentation of grape musts. Yeast.

[37] Velázquez R., Zamora E., Álvarez M.L., Álvarez M.L., Ramírez M. (2016). Using mixed inocula of new killer strains of *Saccharomyces cerevisiae* to improve the quality of traditional sparkling-wine. Food Microbiol..

[38] Maqueda M., Zamora E., Rodríguez-Cousiño N., Ramírez M. (2010). Wine yeast molecular typing using a simplified method for simultaneously extracting mtDNA, nuclear DNA and virus dsRNA. Food Microbiol..

[39] E.C. Nº 761 (1999). Amending Regulation EEC Nº 2676/90 determining community methods for the analysis of wines. Off. J. Eur. Community.

[40] Ambrona J., Vinagre A., Ramírez M. (2005). Rapid asymmetric evolution of *Saccharomyces cerevisiae* wine yeasts under apparently non-selective conditions. Yeast.

[41] Ramírez M., Vinagre A., Ambrona J., Molina F., Maqueda M., Rebollo J.E. (2004). Genetic instability of heterozygous hybrid populations of natural wine yeasts. Appl. Environ. Microbiol..

